# [Retracted] Semaphorin 5A suppresses the proliferation and migration of lung adenocarcinoma cells

**DOI:** 10.3892/ijo.2026.5894

**Published:** 2026-05-18

**Authors:** Pin-Hao Ko, Govinda Lenka, Yu-An Chen, Eric Y. Chuang, Mong-Hsun Tsai, Yuh-Pyng Sher, Liang-Chuan Lai

Int J Oncol 56: 165-177, 2020; DOI: 10.3892/ijo.2019.4932

Following the publication of the above paper, it was drawn to the Editor's attention by a concerned reader that Fig. 1, showing that the downregulation of SEMA5A in lung adenocarcinoma tissues is associated with poor overall survival, was strikingly similar to a figure that had previously appeared in an article by Dan Wang and colleagues in *American Journal of BioMedicine* in 2014 (doi: 10.18081/2333-5106/014-03/292-305). Upon receiving notification of this issue, the authors identified that Fig. 1, as shown, had been included in this paper erroneously, and requested that a corrigendum be published, featuring the correct data for Fig. 1. However, after carefully considering the matter, the Editor of *International Journal of Oncology* has decided that this paper should instead be retracted from the Journal on account of a lack of confidence in the presented data. The Editor apologizes to the readership for any inconvenience caused.

